# Corporate governance data of 6 Asian economies (2010–2017)

**DOI:** 10.1016/j.dib.2018.07.048

**Published:** 2018-07-27

**Authors:** Raja R.R. Singareddy, Shabana Chandrasekaran, Balamurugan Annamalai, Pratyush Ranjan

**Affiliations:** XLRI Jamshedpur, India

## Abstract

This article contains firm-level data on 1) aggregated corporate governance score, and 2) financial data for the period 2010–2017. The study includes 626 companies from 6 Asian countries: China, India, Indonesia, Japan, South Korea and Thailand. Aggregated corporate governance score is calculated using 13 firm-level attributes: board size, board independence, CEO duality, board meeting attendance, independence of audit committee, auditor ratification, independence of compensation committee, independence of nomination committee, shareholder-approved poison pill, dual class unequal voting rights of common shares, staged board, diversity of board and board duration. Finally, six firm-level financial data are included.

**Specifications Table**

**Table:** 

Subject area	*Finance*
More specific subject area	*Corporate finance, Corporate governance*
Type of data	*Excel file*
How data was acquired	*Bloomberg database*
	*Thomson Reuters database*
	*Prowess-CMIE database*
Data format	*Filtered, Processed*
Experimental factors	*The firm sample is based on the listing in the stock exchange-index of six Asian countries.*
Experimental features	*Cross-sectional data of two measures – Corporate Governance attributes and Financial data for the period 2010–2017.*
Data source location	*China, India, Indonesia, Japan, South Korea and Thailand*
Data accessibility	*Data is within this article*
Related research article	*A relevant research article to the collected data is*[1].

**Value of the data**

• The dataset incorporates firms’ aggregated corporate governance score with the financial data.
• The data can be useful to study the evolution of corporate governance in the Asian context, both at the firm level as well as country level. The research could benefit to empirically assess and compare the impact of various regulatory measures in different countries.
• The raw data can be useful for researchers to investigate different corporate governance attributes and firms’ performance.
• The dataset provides aggregated corporate governance score that has broad financial applications and can be appended with other critical financial indicators.

## 1 Data

The cross-sectional data consist of 5008 year-observations of 626 firms recorded in Bloomberg database during 2010–2017. Further, we retrieved data from Thomson Reuters database and CMIE-prowess database to consolidate the available information across databases. Data on country-wise firm count is shown in Table 1. Each observation recorded aggregated corporate governance score and six Financial indicators.

**Table 1:** Data descriptive.

**No.**	**Country**	**Exchange – index**	**Firm count**
1	China	Shanghai Stock Exchange (SSE 180)	180
2	India	Bombay Stock Exchange (BSE 100)	101
3	Indonesia	Indonesia Stock Exchange (IDX – LQ45)	45
4	Japan	Tokyo Stock Exchange (TSE 100)	100
5	South Korea	The Korea Composite Stock Price Index (KOSPI 100)	100
6	Thailand	The Stock Exchange of Thailand (SIT)	100

## 2 Experimental design, materials, and methods

We obtained the 13 corporate governance attributes for each firm individually. The attributes were converted into binary measures based on the conversion logic (Table 3), and the aggregated corporate governance score is calculated as in Gompers et al. [1]. Similarly, the financial measures for each firm was calculated. Finally, these two were merged to create the consolidated datasheet.

The firm-level descriptive data recorded are explained in Table 2. Description of corporate governance attributes and financial indicators are shared in Table 3, Table 4 respectively.

**Table 2:** Firm descriptive.

**Variable name**	**Description**
Ticker_Id	Unique identity for the publicly traded company
Indx_Members	Members of stock index of respective countries
Country	Country in which the company is listed
Year	Considered year

**Table 3:** Corporate governance attributes.

**Variable name**	**Data type**	**Description**	**Conversion formula**
Board size	Count	Number of directors on the board	Value greater than or equal to 5 is 1; 0 otherwise
Board independence	Percentage	A board which has a majority of independent directors who are not affiliated with the firm	Value greater than or equal to 50% is 1; 0 otherwise
CEO duality	Binary (Yes/No)	A situation where the CEO also holds the position of chairman of the board	No is 1; 0 otherwise
Board meeting attendance	Percentage	Attendance by the directors of the board for the board meeting	Value greater than or equal to 66.67% (2/3) is 1; 0 otherwise
Independence of audit committee	Percentage	A committee with a majority of non-executive directors that connects board of directors and external auditors (Percentage of independent directors on audit committee).	Value greater than or equal to 50% is 1; 0 otherwise
Auditor ratification	Binary (Yes/No)	A firm appoints an auditor with annual ratification by the shareholders during the annual general meeting	Yes is 1; 0 otherwise
Independence of compensation committee	Count	A committee with a majority of non-executive directors to oversee the executive compensation	Value greater than or equal to 3 is 1; 0 otherwise
Independence of nomination committee	Percentage	A committee which examines board of directors of the firm and evaluates skills needed for directors	Value greater than or equal to 50% is 1; 0 otherwise
Shareholder approved poison pill	Binary (Yes/No)	In the event of a hostile takeover, shareholders are provided with exclusive rights to cancel the deal	Yes is 1; 0 otherwise
Dual class unequal voting rights of common shares	Binary (Yes/No)	This provision limits voting rights of some equity holders while expanding those of others	No is 1; 0 otherwise
Staggered board	Binary (Yes/No)	A board in which replacement of directors is delayed due to overlapping terms and classes	No is 1; 0 otherwise
Diversity of board	Percentage	Women representation in the board of directors	Value greater than or equal to 1 is 1; 0 otherwise
Board duration	Count	Duration of director׳s continuation in the board	Value less than 9 is 1; 0 otherwise
Corporate governance score	Continuous	Sum (aggregated) of the binarised corporate governance attribute value	Value ranges from 0 to 13

**Table 4:** Descriptive of financial indicators.

**Variable name**	**Data type**	**Description**
LogAsset	Real-valued numeric	Natural logarithm of an asset is approximately equal to the rate of return of an asset
CAPEXByAsset	Real-valued ratio	The ratio of CAPEX to Assets
CashByAsset	Real-valued ratio	The ratio of Cash to Assets
DebtByAsset	Real-valued ratio	The ratio of Debt to assets is an indicator of financial leverage.
PPNEByAssets	Real-valued ratio	The ratio of PPNE to Assets
RNDBYAssets	Real-valued ratio	The ratio of Research and Development expenditure to Assets

The statutory requirements in each country vary, as a result, the firms need not disclose all the attributes of corporate governance. Hence, the data for certain attributes is not available.

The dataset has been processed and compiled using Microsoft Excel.
